# New pandemic, old bugs: A case of cimicosis (Bed Bug) in a neglected elderly patient during the coronavirus disease quarantine

**DOI:** 10.1590/0037-8682-0791-2020

**Published:** 2021-01-29

**Authors:** Gerson Dellatorre, Vidal Haddad

**Affiliations:** 1 Hospital Santa Casa de Curitiba, Departamento de Dermatologia, Curitiba, PR, Brasil.; 2 Universidade Estadual Paulista, Faculdade de Medicina, Departamento de Dermatologia, Botucatu, SP, Brasil.

**Keywords:** bed bug, Cimex lectularius, COVID-19

## Abstract

Bed bugs are hematophagous arthropods that can parasitize humans. During the coronavirus disease pandemic, there has been an increase in elderly neglect. A man in his 90s came to the hospital complaining of generalized pruritus. Despite being a dependent patient, he was left alone in a home by his relatives during the pandemic. Examination revealed inflammatory nodules in addition to a live bed bug crawling over his trunk. Identifying a bed bug during consultation is an uncommon feature that can help determine a particular diagnosis. As this case shows, the need for social isolation during pandemics can contribute to elder abuse and neglect.

## INTRODUCTION

**FIGURE 1:**
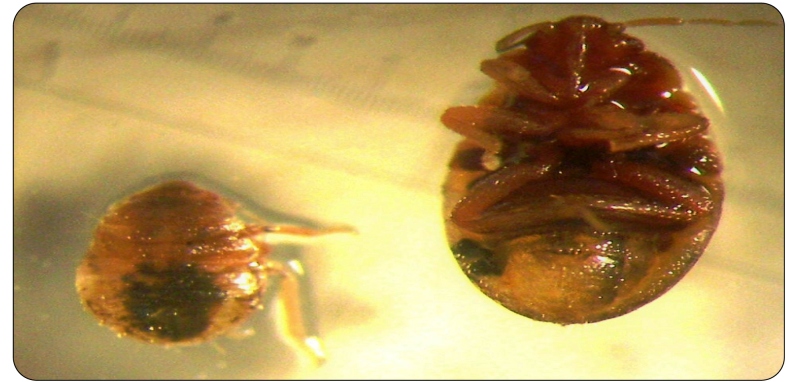
A *Cimex* spp. specimen.

Bed bugs are reddish-brown hematophagous arthropods that parasitize humans as well as other warm-blooded mammals and birds^1^ (Figure 1). They commonly inhabit carpets, cracked floor tiles, bedroom furniture, mattresses, and wall cracks^1^ ^,^ ^2^. Approximately seven different species can parasitize humans, including the most common human bed bug *Cimex lectularius*, distributed worldwide^1^ ^,^ ^2^. During the coronavirus disease (COVID-19) pandemic, there has been a marked increase in reports of elder abuse, including those related to poor hygiene conditions^3^. Herein, we present the case of an older adult (a victim of abuse) with a bed bug infestation during the quarantine period of the pandemic.

## CASE REPORT

A man in his 90s came to the hospital for evaluation of diffuse pruritus that had been present for the past two weeks. Despite being a dependent patient, he was left alone in a home by his relatives; they did not visit him, nor was regular home cleaning undertaken. Food was also mainly by food delivery to ensure social isolation.

**FIGURE 2:**
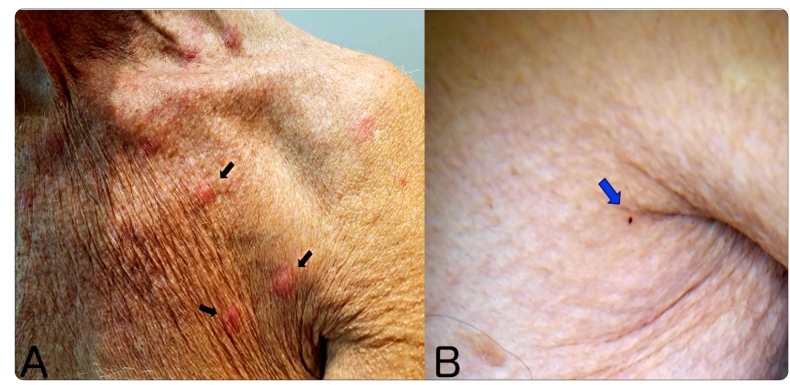
**A:** Erythematous nodules on the patient’s trunk. Note the arrangement where some lesions form a group of three, a few centimeters apart from each other; this is a pattern known as the “breakfast, lunch and dinner” sign (arrows); **B:** A live human bed bug (*Cimex* spp.) crawling over the patient’s trunk (polarized light, 4x magnification).

Physical examination revealed erythematous nodules and excoriations, predominantly on his trunk and arms. Some of the nodules were arranged in a group of three lesions, featuring the “breakfast, lunch, and dinner” sign which identifies flea and bed bug bites (Figure 2A)^2^. A live bed bug, not previously perceived by the patient, was also spotted on his trunk (Figure 2B).

Diagnosis of cimicosis was made; topical corticosteroids and systemic non-sedating antihistamines were administered. All close relatives were informed to help the patient with his basic needs and to arrange for professional home disinfestation.

## DISCUSSION

Accurate diagnoses of skin reactions to insect bites are challenging due to their non-specific clinical manifestations. The “breakfast, lunch, and dinner” pattern of these lesions is characteristic, not just for infestation by bed bugs of *Cimex* genus but also fleas^2^ (Figure 1). Since bed bugs usually seek shelter during the day and become inactive while digesting their blood meal, direct observation of a live bedbug during a consultation (as shown in the image sequence) is an uncommon feature^1^.

Although topical corticosteroids, oral antihistamines, and good personal hygiene are usually enough to control cutaneous symptoms, bed bugs are very resourceful insects. Adults may live up to a year without feeding, making them difficult to eliminate; they usually require a professional pest control service^2^. In Brazil, there are few reports about bed bug infestations the literature. Nowadays, bed bugs are considered uncommon in the industrialized world; furthermore, anecdotal reports suggest that bed bugs are increasingly common in the Western world^3^.

Elder abuse can be physical, emotional, financial, or any combination of these^4^. As shown in this case, the need for social isolation can contribute to elder abuse in the form of neglect by family members. The rights of the elderly are secured by laws in many countries. In cases of severe or persistent neglect, healthcare professionals must also inform local authorities, to protect the patient.
